# Comparative transcriptome analysis of dioecious floral development in *Trachycarpus fortunei* using Illumina and PacBio SMRT sequencing

**DOI:** 10.1186/s12870-023-04551-x

**Published:** 2023-11-03

**Authors:** Feng Xiao, Yang Zhao, Xiurong Wang, Yuexiong Mao, Xueyan Jian

**Affiliations:** 1 Institute for Forest Resources and Environment of Guizhou, Key Laboratory of Forest Cultivation in Plateau Mountain of Guizhou Province, College of Forestry, Guizhou University, Guiyang, 550025 Guizhou China; 2 School of Continuing Education, Yanbian University, Yanji, 133002 Jilin China

**Keywords:** *Trachycarpus fortunei*, Floral development, PacBio SMRT, Transcriptome, TO-GCN

## Abstract

**Background:**

*Trachycarpus fortunei* is a plant with significant economic and ornamental value. Both male and female flowers of *T. fortunei* originate as bisexual flowers, and selective abortion occurs during floral development. However, the regulatory mechanisms underlying this process remain unclear in *T. fortunei*. In this study, transcriptome sequencing with Illumina and Pacific BioSciences (PacBio) single-molecule real-time (SMRT) platforms were used to investigate gene expression differences between male and female *T. fortunei* plants.

**Results:**

A total of 833,137 full-length non-chimeric (FLNC) reads were obtained, and 726,846 high-quality full-length transcripts were identified. A total of 159 genes were differentially expressed between male and female flowers at all development stages. Some of the differentially expressed genes (DEGs) showed male bias, including serine/threonine-protein kinase (*STPK*), *THUMP1 homolog* and other genes. Through single-nucleotide polymorphisms(SNPs) identification, 28 genes were considered as potential sex-associated SNPs. Time-Ordered Gene Co-expression Network (TO-GCN) analysis revealed that *MADS2* and *MADS26* may play important roles in the development of female and male flowers *T. fortune* plants, respectively.

**Conclusions:**

These findings provide a genetic basis for flower development and differentiation in *T. fortunei,* and improve our understanding of the mechanisms underlying sexual differentiation in *T. fortunei*.

**Supplementary Information:**

The online version contains supplementary material available at 10.1186/s12870-023-04551-x.

## Introduction

*Trachycarpus fortunei* (Hook.) H. Wendl. (Fam.*: Arecaceae*) is a type of evergreen tree commonly known as the "mountain palm". Its unopened flower buds ("brown fish") and hearts of palm are edible [1], whiles its fiber can be used as a composite material [2]. *T. fortunei* has important ornamental value. *T. fortunei* is a dioecious plant; both male and female flowers originate from bisexual flowers. The female flower is composed of three functional pistils and six companion stamens on the side; the companion stamens selectively abort, and the female flower becomes a unisexual flower. The male inflorescence has two to three branches, consisting of six functional stamens and three associated pistils on the inner side; with the selective abortion of pistils, the male flower becomes a unisexual flower [3].

Recent research has shed light on the hormone response pathways involved in the identification of sex-determining genes, specifically the cytokinin (CK) and ethylene response pathways. These pathways have been independently proven multiple times to control sex [4]. During the development of male and female flowers in *T. fortunei*, the levels of indole-3-acetic acid (IAA), abscisic acid (ABA), and trans-Zeatin-riboside (ZR) are higher in female flowers than in male flowers at corresponding stages; the higher concentration of IAA, ABA, and ZR is thought to facilitate the development and maturity of female flowers [5]. Conversely, *cytokinin dehydrogenase 6* (*CKX6*) was found to be up-regulated in male flowers of *T. fortunei*, suggesting that a lower concentration of CK is more conducive to male flower development [6]. In dioecious species, the transition from hermaphroditism to parthenogenesis is controlled by sex-determining genes [7]. A typical dicot flower is divided into four parts: petals, sepals, pistils, and stamens. Floral MADS-box genes are well known for their significant role in flower development [8]. Based on further studies on flower development, the ABC model [9] was extended to the ABC(D)E quaternary model [10]. During organogenesis, the ABC homeotic gene seems to control the rate and orientation of cell divisions [11]. Different floral organ identities are regulated by various gene combinations, namely, A + E (sepals), A + B + E (petals), B + C + E (stamens), C + E (carpels), and D + E (ovules) [10]. Only male flowers develop when B-class genes are normally expressed, and only female flowers appear if B-class genes are repressed [12]. Among the 416 angiosperm families, *Arecaceae* are striking in possessing almost all possible combinations of hermaphroditic and/or unisexual flowers [13]. Thus, studying *T. fortunei* flower inflorescence and flower body development is crucial for understanding the evolutionary relationship between the palm family and other angiosperm families [14].

Third-generation Pacific BioSciences (PacBio) single-molecule real-time (SMRT) sequencing does not need to interrupt RNA fragments, and direct reverse transcription can be used to obtain full-length cDNA, which can generate long reads of up to 60 kb, with half of them being longer than 20 kb [15–17]. PacBio SMRT has revolutionized transcriptome-based studies of candidate genes in key pathways and gene regulation in non-model organisms [18]; for instance, it can provide accurate genetic information for flower color development [19], sex determination [20]*.* In order to clarify the genes related to sex differentiation/regulation during the development of male and female flowers in *T. fortunei*, anatomical and phenological observations were carried out, a comprehensive transcriptome of *T. fortunei* was constructed by combining PacBio SMRT and Illumina sequencing. This study will help us understand the development process and sex-determination mechanism of *T. fortunei*, providing a valuable genetic resource for molecular-assisted breeding.

## Materials and methods

### Test materials

**Fig. 1:**
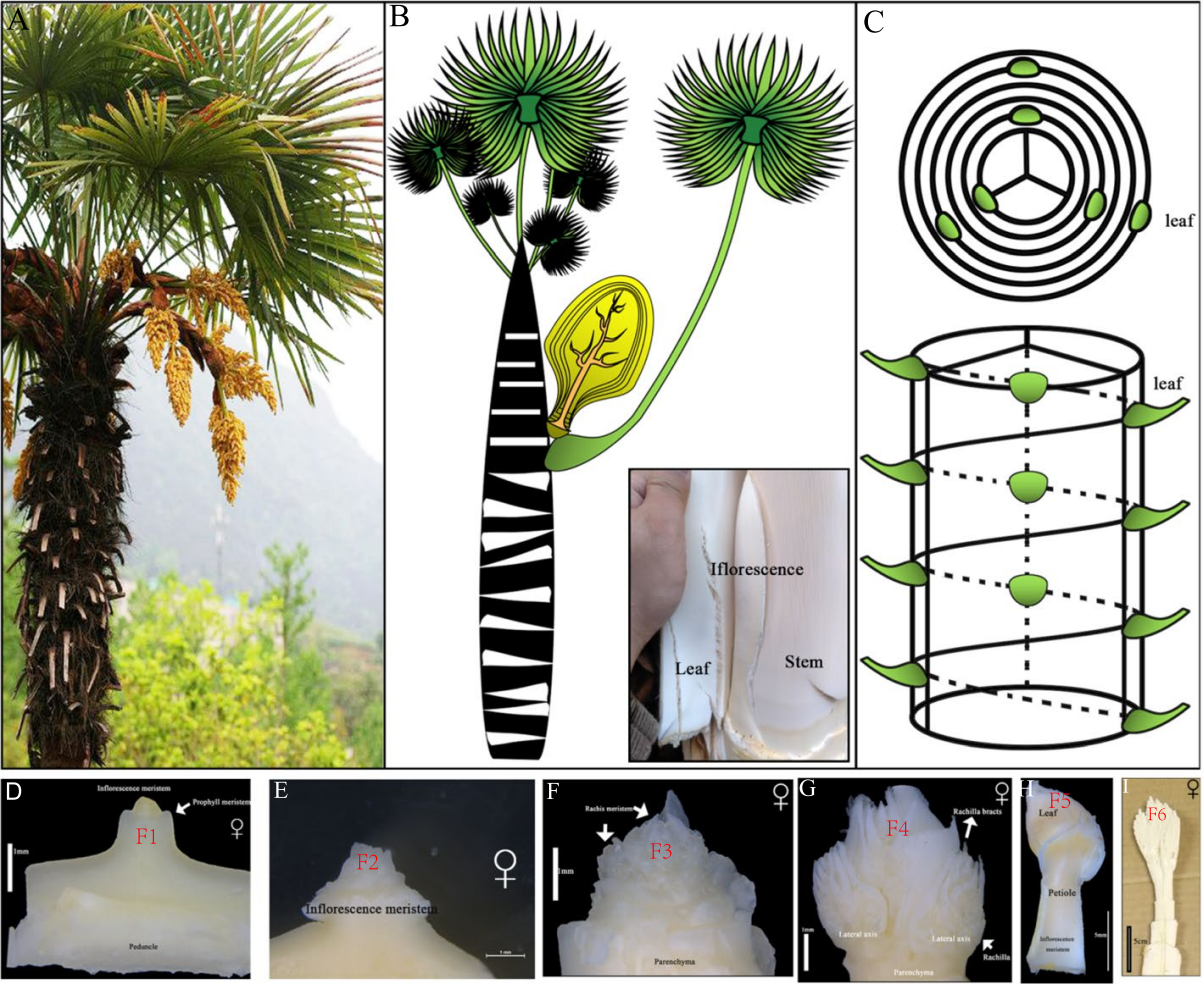
Schematic diagram of sample collection **A** *T. fortunei* plant in natural state. **B** Manual diagram of female plant morphology. **C**: Position of leaf and inflorescence. **D** Top meristem of female *T. fortunei* (F1). **E** Flower primordium initiation (F2). **F** Inflorescence primordium (F3). **G** Spike axis period (F4). **H** The single flower form (F5). **I** Female flower registration, degradation (F6)

Materials were collected from an artificially planted *T. fortunei* forest located in Guiding County, Guizhou Province, China. *T. fortunei* has a single stem with a palmate petiole and petiole base that wraps around the stem (Fig. 1A). Ten female and ten male *T. fortunei* of 10 years old were selected, and the morphological and structural organization of male and female inflorescences were observed. The inflorescence opening status was observed every day, and the time and characteristics of significant changes, such as inflorescence emergence, bract breakthrough, peduncle elongation, and inflorescence flowering, were recorded. The initial flowering stage was defined as when the number of open flowers on a single inflorescence was less than 5%; the full flowering stage was defined as when the number of open flowers was greater than 50%; the final flowering stage was defined as when the number of open flowers was greater than 95%. The tightly wrapped leaf sheath fibers were peeled off the *T. fortunei* tree trunk layer by layer from the bottom to the top, and the complete inflorescence (the smaller inflorescence has a slight petiole) was cut to the meristem at the top of the stem. The inflorescence length, flower morphological characteristics, and other measurements were taken using a scale. The length of the inflorescence axis was measured and, and the number of visibly separated branches was recorded. The number of flowers was measured by weighing 100 individual flowers, which was biologically repeated three times. A total of 30 male and 30 female individual flowers that were fully bloomed and free from pests and diseases were randomly selected. Each flower part was separated and dissected under a stereomicroscope, and pictures were taken using an intelligent stereofluorescence microscope. The diameter of the flowers, the length and width of the sepals and petals, the length of the stamen and anther, and the diameter of the ovary and ovule were measured. Stamens and pistils were collected from the male and female flowers of the *T. fortunei* for microstructure analysis using scanning electron microscopy (S-4800, Hitachi *Ltd.*, Japan).

For the PacBio SMRT sequencing, to cover the genetic information of *T. fortunei* as much as possible, samples were collected from two different parts of 10-year-old male and female *T. fortunei* as materials, including new leaves, old leaves, bracts, flower stalks, apical meristems, and different stages of flower organ development (inflorescence primordium initiation, inflorescence meristem initiation, spikelet axis stage, single flower morphogenesis stage, and female/male flower stagnation and degeneration stage).

For the Illumina sequencing, three 10-year-old female and male *T. fortunei* plants were selected, respectively; the samples were collected from the female and male plants at different stages of flower organ development. In the female plants, the tissues included the top meristem (Fig. 1D, labeled F1). Flower primordium initiation (Fig. 1E, labeled F2), inflorescence primordium (Fig. 1F, labeled F3), spike axis period (Fig. 1G, labeled F4), single flower formation (Fig. 1H, labeled F5), female flowers registration, degradation (Fig. 1I, labeled F6), and the morphology of the samples can be found in Fig. 1. Similarly, different parts of the male plants were collected and labeled as M1-M6. All the samples were collected on a morning between 9:00 and 10:00 AM. There were three biological repeats in each group, which were then washed with distilled water, followed by quick freezing with liquid nitrogen and storage at -80 ℃.

### Test methods

#### PacBio library construction, sequencing and annotation

RNA integrity was assessed by agarose gel electrophoresis; RNA purity (OD_260/280_ and OD_260/230_), concentration and integrity number (RIN) and 28S/18S were detected using a NanoDrop 2000 spectrophotometer and an Agilent 2100 (Agilent Technologies, Santa Clara, California, USA). The RNA extraction quality and concentration of all samples were satisfactory (A260/280 = 2.0–2.2; A260/230 = 1.8–2.2; 28S/18S = 1.4–2.7; Rin ≥ 8.0). The mRNA was enriched with Oligo (dT) magnetic beads. After all RNA samples were qualified, total RNAs from each sample were mixed and isolated. After the mixed mRNA was qualified, a library was constructed. The sequencing library construction process is following SMARTer™ PCR cDNA Synthesis Kit (company, city, nation). After the library was qualified, it was sequenced base on the PacBio platform (Pacific Biosciences, Menlo Park, CA, USA). The raw bam file was deposited in the Genome Sequence Archive [21] in the National Genomics Data Center [22], China National Center for Bioinformation/Beijing Institute of Genomics, Chinese Academy of Sciences (GSA: CRA009442) and is publicly accessible at https://ngdc.cncb.ac.cn/gsa.

The raw sequencing subreads from the PacBio platform were filtered using SMRTLink v5.0 (https://www.pacb.com/support/software-downloads/) with default parameters. The circular consensus sequence (CCS) was obtained by merging the subreads from the same polymerase reads, using self-correction. Full-length Non-concatemer (FLNC) sequences are a kind of CCS that contains both 5’ and 3’ primers and a poly-A tail, without chimeric reads. After clustering the FLNC reads using the IsoSeq module, the sequences consistent across each cluster were further corrected, and finally HQ (high-quality full-length transcripts with accuracy greater than 99%) and LQ (low-quality) transcripts were obtained, respectively. CD-HIT [23] was used to remove redundant sequences in transcripts. BUSCO [24] estimated the integrity of the transcripts of some conserved genes in related species. According to the priority order of NR (NCBI non-redundant protein sequence), Swiss-Prot and KOG (clusters of euKaryotic Orthologous Groups), the transcripts were aligned to the above protein library (evalue < 0.00001), while ESTScan [25] was used to predict the coding region (sequence direction 5'- > 3') if none of the above protein databases were compared. To accurately predict lncRNAs (long noncoding RNAs), the Coding Potential Assessment Tool (CPAT), Coding–Non-Coding Index (CNCI), Coding Potential Calculator (CPC) and Pfam protein structure domain analysis (Pfam) [26–29] were used to predict the coding potential of transcripts.

#### Illumina transcriptome library preparation and sequencing

The mRNA was enriched with Oligo (dT) magnetic beads. The mRNA was added to a fragmentation buffer and cut into short fragments. Using mRNA as a template, cDNA was reverse-transcribed using six-base random primers. The preparation and procedure of the cDNA library were referenced from previous protocols [30]. After the libraries passed the quality test, qualified libraries were sequenced using the Illumina Novaseq 6000 platform with paired-end 150 bp reads. The raw reads generated from sequencer were deposited in the NCBI SRA database (accession BioProject: PRJNA928793).

### DEGs identification and enrichment

To identify gender-related loci and obtain broader gene expression patterns, 12 public transcriptome datasets (*acc.*: SRR10120876-SRR10120887) [6] were downloaded and analyzed together. The female flower sample and female leaf sample were labeled as F7 and FL, respectively; male flower samples and leaves samples were labeled as M7 and ML, respectively. fastp v0.22.0 [31] was used for the quality control of raw data. The PacBio SMRT transcriptome was used as the reference transcriptome. Paired-end reads from libraries were aligned to the *T. fortunei* reference transcriptome by bowtie2 v2.4.3 (https://bowtie-bio.sourceforge.net/bowtie2) [32]. The quantification of gene expression levels were estimated by the fragments per kilobase of transcript per million fragments mapped (FPKM) using RSEM v1.2.12 (https://github.com/deweylab/RSEM) [33]. Principal component analysis (PCA) was performed using the expression levels of all samples. Pairwise gene expression comparisons were performed using the DESeq2 v1.40.1 [34]. The differentially expressed genes (DEGs) screening threshold was set to *p-value* < 0.05 and |foldchange|> 2. In order to find the transcriptomic single-nucleotide polymorphisms’ (SNPs) loci in the male and female *T. fortunei*, BCFtools v1.15.1 (https://github.com/samtools/bcftools) and samtools v1.15.1 (http://samtools.sourceforge.net/) were used to map the clean reads of each male and female individual to the reference transcripts to identify potential sex-associated candidates of transcripts. To obtain a more solid result, more stringent cutoff values were applied, as follows [35]: (1) all unique reads in each individual of the same gender must cover the same nucleotide position; (2) the genes must be polymorphic (in terms of SNPs); (3) the SNPs must be common in each sex type, for instance, males shared one SNP and females shared the other one. The SNPs that satisfied the above criteria were considered as potential sex-associated SNPs, and the corresponding genes were identified as putative sex-associated genes. The clusterProfiler v4.8.1 Package [36] was used to perform Gene Ontology (GO) and Kyoto Encyclopedia of Genes and Genomes (KEGG) enrichment. The Short Time-series Expression Miner (STEM) [37] software (https://www.cs.cmu.edu/%7Ejernst/stem/) was used to perform the trend cluster analysis which was divided into seven stages according to the part of occurrence and sex; combinations of different stages of combined genes were input and normalized, the number of models was set to 50, and the gene sets of each profile were selected for GO and KEGG enrichment.

### Time-ordered gene co-expression networks (TO-GCNs)

To investigate the regulatory mechanisms of flower formation, a recently developed method of reconstructing time-ordered gene co-expression networks (TO-GCNs) [38] was used. Eight GCN co-expression types under two genders (C1 and C2): C1 + C2 + , C1 + C20, C1 + C2-, C10C2 + , C1-C2 + , C1-C2-, C1-C20, and C10C2-, where + , - , 0 indicate positive, negative, and no co-expression, respectively [39]. TO-GCNs for female flowers (female-specific TO-GCN, C1 + C20) and male flowers (male-specific TO-GCN, C10C2 +) were constructed, as well as a consensus TO-GCN (C1 + C2 +) between the two networks. The Pearson correlation coefficient (PCC) cutoff value was 0.84 between structural genes and transcription factor (TF)s. The seeds, around which the gene clusters were based, required these genes to be highly expressed at the control (term1) and down-regulated at all subsequent time points. The Teosinte branched1/Cycloidea/Proliferating cell factor (*TCP*) gene (i1_HQ_Tf_c85292_f2p0_1223) was selected as the seed. The network visualization was drawn by the Crosslink v0.1.0 Package [40].

### Statistical analysis

Significant differences were calculated using a one-way ANOVA with a least significant difference test (LSD) and a significance level set at *p* ≤ 0.05 using the R v4.2.3 software (https://www.r-project.org/) [41]. A radar chart was drawn by the see Package v0.8.0 [42]. Principal component analysis (PCA) was performed using the prcomp function of the stats v3.6.2 Package. Cluster analysis of the samples was performed using the hcluster function and visualization was performed using the factoextra v1.0.7 Package [43].

## Results

### Male and female flowering periods and flower morphological characteristics

**Fig. 2:**
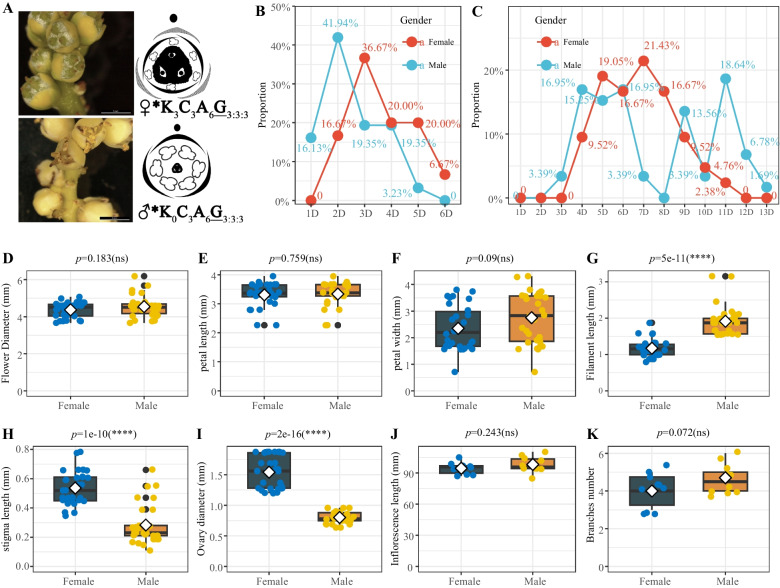
Observation of duration of flowering period and characteristics of male and female flowers of *T. fortunei.* **A** Phenotype and flower formula of male and female flowers. **B** Single flowering time of female and male flowers. **C** Female and male inflorescence time. **D** Diameter of flowers. **E** Petal length. **F** Petal width. **G** Stigma length. **H** Stigma length. **I** Ovary diameter. **J** Inflorescence length. **K** Branches number. *Note:* In **D**-**K**, t-test was performed by using the R-function t.test. "*" Sign indicates that there is difference between the two groups, *p < 0.05, **p < 0.01, ***p < 0.001, ****p < 0.0001, and "ns" means no difference

According to the statistics on the characteristics and duration of single flower florescence, it was observed that during the early stage of male flower development, there were three yellow-green calyxes. As the flowers matured, they gradually degenerated (some individuals were able to partially retain the flowers). At this stage, six stamens would expand, while three aborted pistils would separate (Fig. 2A). The single male flower would last for 1–5 days, with the highest frequency being 2 days (41.94%). On the other hand, the single female flower would last for 2–6 days, with the highest frequency of appearance being 3 days (36.67%) (Fig. 2B).

The duration of the male inflorescence was 3–13 days, with an average of 7.4 ± 3.0 days, while the duration of the female inflorescence was 4–11 days (Fig. 2C). The diameters of the female and male flowers did not show any significant difference (Fig. 2D), and the lengths and widths of petals were similar in size (Fig. 2E–F). However, the filament length of female flowers was significantly shorter than that of male flowers ( Fig. 2G). Additionally, the lengths of the female flowers’ stigmas and ovaries were significantly longer than those of males (Fig. 2H and I). There was no significant difference in the inflorescence length (Fig. 2J) and branches number (Fig. 2K).

### Acquisition of high-quality PacBio SMAT transcriptome of *T. fortunei*

**Fig. 3:**
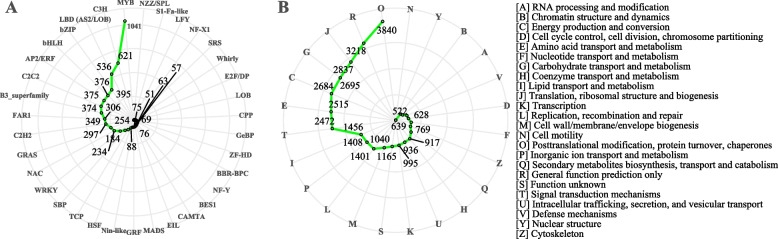
Annotation classification of transcripts. **A** Prediction of TFs. **B** Prediction of COGs. *Note:* In **B**, the terms that number over 500 were selected to showed the number size; the right-hand area shows the letter abbreviations and corresponding spellings

The mixed samples of the *T. fortunei* were sequenced using the PacBio SMRT platform. A total of 14,776,542 subreads (46 Gb of bam files) were obtained, the max read was 153604 bp, the mean read was 1704 bp. After quality control, 236,267 isoforms were obtained, consisting of 36,824 HQ transcripts and 199,443 LQ transcripts. The mean length of the HQ isoforms was 2,102 bp. A total of 143,857 transcripts encoded by 136,825 genes were obtained after clustering and removing redundancy by CD-HIT. The mean length of the final consensus transcripts was 2,202 bp. The analysis of transcriptome completeness with BUSCO showed that a total of 287 (94.7%) genes were complete, 4 (1.3%) were fragmented and 12 (4%) were missing BUSCOs. Four tools were used to identify the lncRNA from the transcriptome, A total of 6,574 LncRNAs were identified. Functions annotations showed that 130,974 (91.04%) transcripts were successfully matched to known sequences at least one database. The identification of TFs showed that the size of the MYB family was the greatest (1041) and the size of the MADS box gene family was 69 (Fig. 3A); the full-length transcript was annotated for COG function, and the term “Posttranslational modification, protein turnover, chaperones” had the highest number of matches (Fig. 3B).

### Comparison of transcriptome and analysis of DEGs

**Fig. 4:**
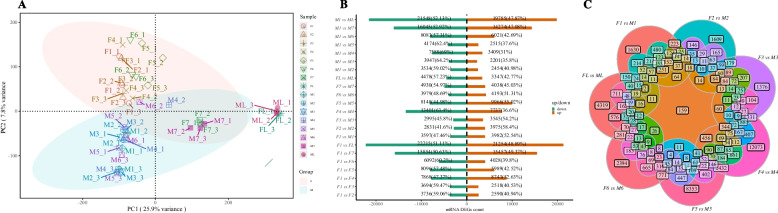
Analysis of transcriptome data and number distribution of DEGs. **A** PCA analysis of all samples. **B** Distribution of DEGs numbers between the different groups. **C** Venn diagram of DEGs between the different groups. Note: In **A**, different colors and shapes represent different groups, and the two ellipses represent the 95% confidence interval of the distribution between different sex types

The Q20 and Q30 scores for all samples ranged from 98.37% to 98.89% and 95.12% to 96.12%, respectively. The error rate ranged form 0.0119 to 0.0238% and the average content of GC was around 47.64%, the four base content distribution of each sample were relatively uniform, indicating high quality and accuracy of the high-throughput sequencing [44]. The mapping results show that the mapped ratios of all samples were between 81.16% and 87.11%, indicating that the constructed PacBio SMRT transcriptome had high quality and covered most of the gene information, making it suitable for using as a background transcriptome for further analysis. After calculating the expressions of all samples, the PCA analysis (Fig. 4A) showed that the correlation between samples in the same group was high, and the relative PCA distance was close, demonstrating excellent repeatability. Additionally, the relative PCA distance between samples of different sexes in the same period was large.

Pairwise differential expression analysis was performed using the criterion of |log_2_(FoldChange)|> 1, resulting in 7579 DEGs in the F1 vs. M1 comparison contained 3982 (52.54%) of them were upregulated and 3597 (47.46%) of them were downregulated, 6806 DEGs in the F2 vs. M2 comparison contained 3975 (58.4%) of them were upregulated and 2831 (41.6%) of them were downregulated, and 6540 DEGs in the F3 vs. M3 comparison contained 3545(54.2%) of them were upregulated and 2995 (45.8%) of them were downregulated (Fig. 4B). A total of 159 genes were differentially expressed between male and female flowers at all development stages (Fig. 4C). Moreover, through SNP identification, there were 28 genes that have sex-related SNP sites (Table S1), which can be used as gender markers to distinguish the sex of *T. fortunei*. Among them, seven genes were always differentially expressed in male and female plants; these were *ATP-citrate synthase beta chain protein 1* (i2_LQ_Tf_c201692_f1p1_2479), *Probable methyltransferase PMT7* (i2_LQ_Tf_c179993_f1p0_2878), *Triacylglycerol lipase SDP1* (i3_LQ_Tf_c2184_f1p0_3572), *THUMP domain-containing protein 1*(i4_LQ_Tf_c2911_f1p0_4933), *Ubiquitin carboxyl-terminal hydrolase 24* (i2_LQ_Tf_c153712_f1p0_2199), *Protein ecdysoneless homolo*g (i2_HQ_Tf_c270038_f10p1_2238), and *SUPPRESSOR OF ABI3-5* (i3_LQ_Tf_c17299_f1p0_3046).

The GO enrichment (biological process (BP) category) analysis has revealed that DNA replication initiation (GO:0006270) and trichome branching (GO: 0010091) were enriched in the F1/M1 group (Fig. S1A). Trichome branching (GO:0010091), the regulation of photoperiodism, flowering (GO:2000028), jasmonic acid mediated signaling pathway (GO:0009867), red, far-red light phototransduction (GO:0009585) and other terms were enriched in the F2/M2 group. S-adenosylhomocysteine catabolic process (GO:0019510), one-carbon metabolic process (GO: 0006730) and other terms were enriched in the F3/M3 group. Positive regulation of transcription by RNA polymerase II (GO:0045944) was enriched in the F4/M4 group. Cytokinin metabolic process (GO:0009690), pectin catabolic process (GO:0045490), response to auxin (GO:0009733) and other terms were enriched in the F5/M5 group. DNA replication initiation (GO:0006270), s-adenosylhomocysteine catabolic process (GO:0019510), regulation of photoperiodism, and flowering (GO:2000028) were enriched in the F6/M6 group. Regulation of flower development (GO:0009909) was enriched in the F7/M7 group. The GO enrichment (BP category) revealed that the cytokinin metabolic process (GO:0009690), auxin-activated signaling pathway (GO:0009734), cytokinin biosynthetic process (GO:0009691) and other terms were enriched in the F1/F2 group (Fig. S1B). Metal ion transport (GO:0030001), auxin-activated signaling pathway (GO: 0009734), cell population proliferation (GO:0008283) and other terms were enriched in the M1/M2 group (Fig. S1C).

### STEM analysis

**Fig. 5:**
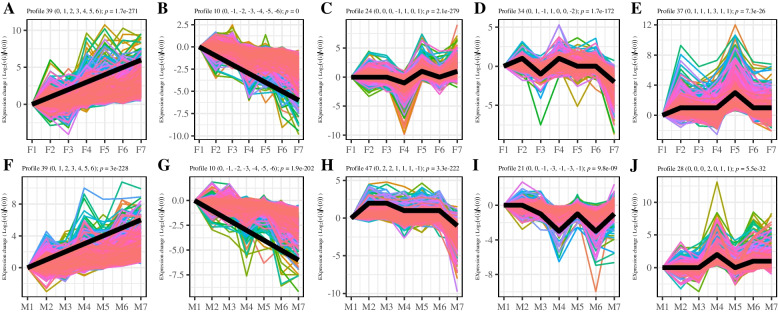
Cluster analysis of the gene expression trend. Note. **A**-**E** Part significant profiles of the expressions of DEGs from the different parts of female flowers. **F**-**J** Part significant profiles of the expression hcluster of DEGs from the different parts of male flowers. Black line represents the profile trendline

The STEM analysis revealed that a total of 13 significant difference profiles were obtained at different developmental stages of female flowers, of which Profile 39 (0, 1, 2, 3, 4, 5, 6) showed a gradual increase in expression with time (Fig. 5A). Hydrogen peroxide catabolic process (GO:0042744), trehalose biosynthetic process (GO:0005992), cell cycle arrest (GO:0007050), leaf development (GO:0048366) and others term were enriched. KEGG enrichment analysis showed that Tryptophan metabolism (ko00380) and Brassinosteroid biosynthesis (ko00905) were enriched. Cyclin-dependent kinase inhibitor (i1_LQ_Tf_c165000_f1p0_1204, i1_LQ_Tf_c382200_f1p0_1019, i1_LQ_Tf_c52295_f1p0_1172) was enriched in the term of cell cycle arrest. KUODA1 (KUA1) (i0_HQ_Tf_c3423_f2p0_735, i1_HQ_Tf_c141330_f13p2_1475), Scarecrow-like protein 23 (i1_HQ_Tf_c311658_f11p1_1695, i1_LQ_Tf_c120676_f1p1_1759), Cytochrome p450 (i1_LQ_Tf_c369423_f1p0_1501, i1_LQ_Tf_c6015_f1p0_1757) and AP2 ERF and B3 domain-containing (i1_LQ_Tf_c74362_f1p0_1296) were enriched in the GO:0048366 term. Profile 10 (0, -1, -2, -3, -4, -5, -6) gradually decreased over time (Fig. 5B). Gene silencing by RNA (GO:0031047), phosphatidylserine biosynthetic process (GO:0006659), multicellular organism development (GO:0007275), small GTPase mediated signal transduction (GO:0007264) and other terms were enriched in this profile.

Profile 24 (0, 0, 0, -1, 1, 0, 1)-related gene expression reached its lowest value at the F4 stage (Fig. 5C). Intramolecular transferase activity, phosphotransferases (GO:0016868), succinate-semialdehyde dehydrogenase [NAD(P) +] activity (GO: 0009013), sucrose synthase activity (GO:0016157), and alpha-amylase activity (GO:0004556) were enriched, while profile 34 (0, 1, -1, 1, 0, 0, -2)-related gene expression peaked at the F4 stage. Ribosome biogenesis (GO:0042254), nucleosome assembly (GO: 0006334), chromosome condensation (GO:0030261), protein demethylation (GO:0006482), carotene catabolic process (GO: 0016121), and xanthophyll catabolic process (GO:0016124) were enriched (Fig. 5D). Profile 37 (0, 1, 1, 1, 3, 1, 1)-related gene expression reached its highest value at the F5 stage (Fig. 5E), wherein proton export across plasma membrane (GO: 0120029), auxin homeostasis (GO: 0010252), regulation of photoperiodism, and flowering (GO: 2000028) were enriched.

A total of 11 significant difference modules were obtained at different developmental stages of male flowers. Profile 39 (0, 1, 2, 3, 4, 5, 6) showed a gradual increase in expression with time (Fig. 5F). Inositol biosynthetic process (GO:0006021), phospholipid biosynthetic process (GO:0008654), and leaf development (GO:0048366) were enriched. *KUA1* (i1_HQ_Tf_c141330_f13p2_1475) and *SCL23* (i1_LQ_Tf_c120676_f1p1_1759, i1_LQ_Tf_c8122_f1p2_1941) were enriched in leaf development (GO:0048366). Profile 10 (0, -1, -2, -3, -4, -5, -6) gradually decreased over time (Fig. 5G). Multicellular organism development (GO:0007275) and gene silencing by RNA (GO:0031047) were enriched. Profile 47 (0, 2, 2, 1, 1, 1, -1) reached its lowest value at the M7 stage (Fig. 5H). Profile 21 (0, 0, -1, -3, -1, -3, -1) reached its lowest value at the M4 and M6 stages (Fig. 5I). Profile 28 (0, 0, 0, 2, 0, 1, 1)-related gene expression peaked at the M4 stage (Fig. 5J), wherein trehalose biosynthetic process (GO: 0005992) and auxin-activated signaling pathway (GO: 0009734) were enriched.

### TO-GCN analysis

**Fig. 6:**
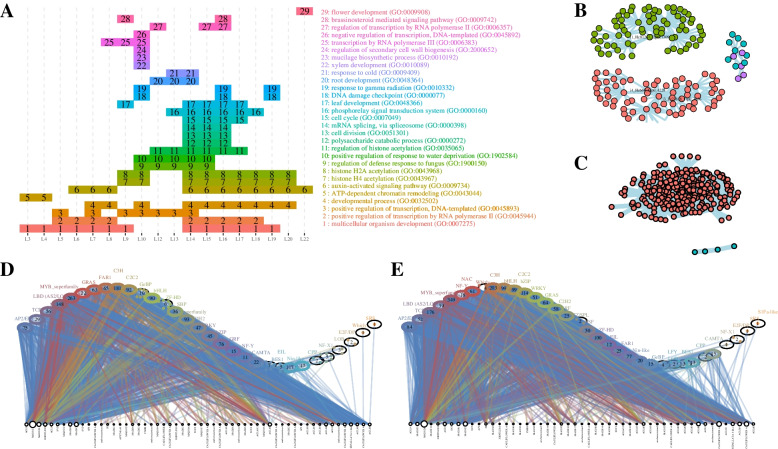
Network alignment of two time-ordered gene coexpression networks (TO-GCN). **A** Overrepresented GO functions at each level. **B** Regulatory subnetwork for GCN10. **C** Regulatory subnetwork for GCN13. **D** Female-special TO-GCN (C1 + C20). **E** Male-special TO-GCN (C10C2 +). *Note*: L1 to L22 indicate the levels identified in the time-ordered gene co-expression network. The black dots with white in the middle represent the MADS-box genes. In D&E, the network node only screened the genes related to MADS box genes

To analyze sex differentiation and determinations in female and male plants of *T. fortunei*, TO-GCNs analysis was implemented on the *T. fortunei* transcriptome with six developmental stages. The analysis of the TO-GCN network with male and female specificity TO-GCN showed that there were 22 consensus modules (C1 + C2 +) in total, which were used to predict the time node order of genes in GCN. If both genes were positively co-expressed in female flowers and male flowers, the two genes were inferred to belong to the C1 + C2 + relationship set, and so GO enrichment analysis (BP category) for genes in each consensus module was conducted (Fig. 6A). Multicellular organism development (GO: 0007275) was enriched in most GCNs, while flower development (GO: 0009908) was only enriched in the L22. The lateral organ boundaries domain (*LBD*) gene (i1_HQ_Tf_c81267_f2p0_1309) and NAC from the transmembrane motif 1 like (*NTM1-like*, i3_LQ_Tf_c23818_f1p0_3636) were the key nodes of L10 (Fig. 6B). i1_HQ_Tf_c7271_f4p0_1338 showed the largest weight value at L13 (Fig. 6C). *MADS2* (i0_LQ_Tf_c12820_f1p0_903) was the main key node of female specialty (C1 + C20) (Fig. 6D). The main key node of male specialty (C10C2 +) was *MADS26* (i0_LQ_Tf_c13201_f1p1_928) (Fig. 6E).

## Discussion

Most flowering plants are hermaphrodites, but around 5 ~ 6% angiosperm species are dioecious, featuring separate unisexual plants bearing either only male or female flowers [45, 46]. The types of flowers can be divided into two categories; type I flowers are bisexual at initiation and become unisexual via the termination of development in one whorl of reproductive organs; type II flowers are unisexual from inception and sex differentiation occurs before the initiation of female or male organ primordia [47]. The leaves on each stem node of the *T. fortunei* are arranged in a spiral pattern, and the inflorescence is located in the center of the petiole base. Each leaf base has only one inflorescence (Fig. 1B and C). Morphological differences between male and female flowers in palms concern size, shape and structural attributes [13]. The inflorescences in the middle and upper parts of the plant opened first, and the lower parts of the inflorescences opened later, showing the characteristics of stage opening. The inflorescence bloomed for 5 to 10 days, showing a "Mass-flowering" pattern. "Mass-flowering" is common in other plants, for instance, *Shorea beccariana* [48] and *Dendrocalamus membranaceus* [49]. Mass-flowering plants can increase bee abundance and ensure pollination [50, 51]. The flowering of a single flower lasts 2 to 4 days (Fig. 2B and C). A single flower of *T. fortunei* is hermaphroditic at the initial stage of stamen and pistil primordium development, and then parthenogenesis occurs due to the selective abortion of stamen and pistil. The stamen stops developing shortly after the differentiation of filaments and anthers, then shrivelled and atrophied, loses cell activity, and does not produce anther wall structure and microspore [52].

As an economically important horticultural plant, there is currently little genetic information available about *T. fortunei*. The PacBio sequencing technique is an efficient approach to obtaining a long sequence, and has been widely applied in plants [53]. In order to obtain comprehensive genetic information on *T. fortunei*, different parts of 10-year-old male and female *T. fortunei* plants were collected for PacBio SMRT sequencing. By sequencing and analysis, a total of 14,776,542 subreads (46 Gb of bam file) were obtained. A total of 236,267 isoforms were obtained, comprising 36,824 HQ transcripts and 199,443 LQ transcripts. On the other hand, a total of 6,574 LncRNAs were predicted based on SMRT sequencing. To obtain the difference in gene expression between female and male *T. fortunei*, comparative transcriptomic analyses were conducted by combining PacBio SMRT and Illumina sequencing technologies. Different stages of floral development (inflorescence primordium initiation, inflorescence meristem initiation, spikelet axis stage, single flower morphogenesis stage, flower stagnation and degeneration stage) were subjected to Illumina sequencing; the mapped ratios of samples ranged from 81.16% to 87.11%, indicating that the constructed PacBio SMRT transcriptome was of high quality. The PCA analysis of the expression between samples showed that there were significant differences between male and female samples (Fig. 4A). Sex-biased genes are thought to be associated with sex determination or sex differentiation [35, 54, 55]. In total, 1946 male-biased genes and 961 female-biased genes were found via the comparative transcriptome analysis in *Spinacia oleracea* [56]. In the sex-determining region of *Populu*s × sib*irica*, both allelic variants of the T-complex protein 1 subunit gamma, *CLC* (Chloride channel protein CLC-c), and *MET1* (DNA-methyltransferase 1) genes were expressed in females [57]. Amongst the 14 floral organ ABCDE model-related genes in *Eucommia ulmoides*, there were 6 (A/B/C/E-class) and 5 (A/D/E-class) genes that displayed male- and female-biased expressions, respectively [58]. Comparative analyses across all 14 species of the *Phoenix* genus (Fam.: *Arecaceae*) identified three potential sex-determining genes, including Y-linked Cytochrome P450 (*CYP703*), glycerol-3-phosphate acyltransferase 6-like (*GPAT3*) and Lonely Guy-like gene (*LOG-like*) [59]. In *E. ulmoides,* genes (*EuAP3*, *EuAG*) with differential expressions at different stages of male and female flower bud development are thought to be associated with sex determination [60]. The persistent differential expression between male and female *T. fortunei* plants may be related to the development of specific organs or sex differentiation. Through the comparative analysis of combinations of male and female flowers at different stages, 159 genes were found to be differentially expressed between male and female flowers all the time (Fig. 4D). Through expression cluster heatmap analysis, it was found that these genes showed obvious biased expressions; serine/threonine-protein kinase (*STPK*), *THUMP1* homolog and others genes were specifically expressed in *T. fortunei* male flowers. The GO enrichment (BP category) revealed that cytokinin metabolic process (GO:0009690), auxin-activated signaling pathway (GO:0009734), cytokinin biosynthetic process (GO:0009691) and other terms were enriched in the F1/F2 group (Fig. S1B). The determination of hormone content in the early flowering period of *T. fortunei* showed that the high contents of IAA, ABA and ZR are more conducive to the development and maturity of female flowers [5]. Early sex identification plays an important role in plant improvement in breeding programs [61–63]. SNP loci can serve as markers for sex differentiation [64]. The partial sequence of the *TBL3* gene of hermaphrodite *Salacca zalacca* revealed ten SNPs compared to those of female and male plants [61]. Through SNP identification between male and female plants, 28 genes were identified as potential sex-associated SNPs (Table S1); for instance, the 1201 sequence position of the male in i2_LQ_Tf_c179993_f1p0_2878 was “A”, and the female was missing ('-'); multiple loci of three genes (i2_LQ_Tf_c46048_f1p0_2640,i1_HQ_Tf_c190705_f3p0_1189, i1_LQ_Tf_c74546_f1p0_1984) exhibited heterozygosity (Fig S2). All the 28 genes can serve as gender markers to distinguish the sex of *T. fortunei* in further research.

STEM analysis can be used to more intuitively predict the functions of genes with the same expression trends. Totals of 13 and 11 significant difference profiles were obtained at different developmental stages of female and male flowers, respectively, of which the module profile 10 (0, -1, -2, -3, -4, -5, -6) gradually decreased over time (Fig. 5B and G) in female and male flowers. Gene silencing by RNA (GO: 0031047) and multicellular organism development (GO: 0007275) were both enriched in the two profiles. On the contrary, there were two profiles that showed a gradual increase in expression with time (Fig. 5A and F). KEGG enrichment analysis showed that Tryptophan metabolism (ko00380) and Brassinosteroid biosynthesis (ko00905) were enriched in the female profile 39. Inositol biosynthetic process (GO: 0006021), phospholipid biosynthetic process (GO: 0008654) and leaf development (GO 0048366) were enriched in the male profile 39. This indicates that the gene sets expressed by male and female flowers may be inconsistent with each other over time.

In order to excavate the modules that are co-expressed and specifically expressed over time, the TO-GCN analysis was conducted. Through the TO-GCN analysis, at the initial stages of flower coloration, *MYB*, *bHLH*, and *WD40* TFs may collectively regulate anthocyanin accumulation in *Rhododendron simsii* [65]. Gene co-expression networks indicated *WRKY* as an essential regulation factor at the early flowering stage [66]. *HmAP1-1*, *HmAP1-2*, *HmAP1-3*, *HmAP2-3*, *HmAP2-4*, and *HmAP2-5* may be related to the development of decorative floret sepals in *Hydrangea macrophylla* [67]. A great majority of these genes encode MADS-box TF; MADS-box plays a crucial role in many important processes in flowering plants. There were 69 MADS-box TFs in *T. fortunei* (Fig. 3A), the systematic phylogenetic tree of all identified MADS-box proteins can be found in Fig. S3. The analysis of the TO-GCN network with male and female specificity TO-GCN showed that there were 22 consensus modules (C1 + C2 +) in total. *MADS2* was the main key node of female-special (C1 + C20) GCN. The main key node of male-special (C10C2 +) GCN was *MADS26*. Zea mays *AGAMOUS1* (ZAG1), *ZAG2* and *MADS2* are not expressed in ear florets in Zea mays mutant branched silkless1–2 (*bd1–2*) (floret development is blocked in the female inflorescence (the ear), whereas florets develop almost normally in the male inflorescence (the tassel), where their expression in tassel florets is similar to that seen in the wild type [68]). *MADS2* in *Volvox* shows female-biased expression [69]. In rice, lodicule identity is mainly specified by the action of *SUPERWOMAN1 (SPW1)* and *OsMADS2* [70]. In *Dacrydium pectinatum,* several MADS-box homologs of the time point-specific unigenes were found to be exclusively expressed in the female reproductive parts in other species, revealing several floral (*MADS2*, *AGL62*, and *LFY*) and hormone biosynthesis and signal transduction genes that could be critical for female cone development [71]. In *Areca catechu*, JA concentration in female flowers was 10 times than that in males on the same inflorescence, JA promotes the development of female flower organs by decreasing the expression of B-function genes, including *AGL16*, *AP3*, *PIb* and *PIc* [72]. By screening for genes significantly correlated with the expression level of *MADS2* (|cor|> 0.5 and *p-value* < 0.01) in the plant hormone signaling pathway, 14 genes that exhibit a positive and significant correlation with *MADS2* expression was identified. This set of genes includes three *GH3* (Gretchen Hagen 3) genes, three *PIF3* (PHYTOCHROME-INTERACTING FACTOR) genes, two *SAUR* (small auxin-up RNA) genes, two *IAA* (auxin-responsive protein) genes. These findings suggest that the regulation of the *MADS2* gene may be influenced by IAA hormones. *MADS2* was the main key node of female-special (C1 + C20) GCN (Fig. 6D), and it is suggested that *MADS2* may play a decisive role in the development of female flowers of *T. fortunei. Oryza sativa OsMADS26* is expressed not only in the roots, but also in the leaves, shoots, panicles, and seeds [10]. Transcript levels of *OsMADS26* were increased in an age-dependent manner in the shoots and roots [73]. Similarly, by screening for genes significantly correlated with the expression level of *MADS26* (|cor|> 0.5 and *p-value* < 0.01) in the plant hormone signaling pathway, 12 genes that exhibit a positive and significant correlation with *MADS26* expression was identified. This set of genes includes five *PP2C* (protein phosphatase 2C) genes, one *STPK* gene. These indicates a potential correlation between *MADS26* and ABA regulation. The main key node of male specialty (C10C2 +) was *MADS26* (Fig. 6E), which implies that *MADS26* may play a decisive role in the development of male flowers of *T. fortunei*.

## Conclusions

This study aimed to identify the genes involved in regulating flower development in *T. fortunei* through anatomical observations and transcriptomic determinations at different stages of flower development. 833,137 FLNC reads and 726,846 high-quality full-length transcripts were obtained. DEGs analysis found that 159 genes were expressed preferentially, while *STPK*, *THUMP1 homolog* and others genes were specifically expressed in *T. fortunei* male flowers. Through SNP identification, 28 genes were determined to be potential sex-associated SNPs. TO-GCN analysis showed that *MADS2* may play a decisive role in the development of female flowers in *T. fortunei,* while *MADS26* may play a crucial role in the development of male flowers.

### Supplementary Information

**Additional file 1: Tab S1.** Hypothetical sex-related genes in *T. fortunei*. POS is the site of the transcript where the sequencespecifc site was located. 

**Additional file 2: Fig. S1.** GO enrichment between different groups. A: Comparison between male and female plant combinations at the same time and position; B: Comparison between different part combinations of female plants; C:Comparison between different part combinations of male plants. Note: the size of the circle represented the gene count, the different colors represented the different groups; the fold enrichment equaled GeneRatio/BgRatio. **Fig. S2.** Sequence diagram of some hypothetical sex-related genes. **Fig. S3. **The systematic phylogenetic tree of all identified MADS-box 

## Data Availability

The raw sequence data of PacBio SMRT have been deposited in the Genome Sequence Archive (Genomics, Proteomics & Bioinformatics 2021) in National Genomics Data Center, China National Center for Bioinformation/Beijing Institute of Genomics, Chinese Academy of Sciences (GSA: CRA009442) that are publicly accessible at https://ngdc.cncb.ac.cn/gsa. The raw reads generated from Illumina sequencing have been deposited in the NCBI SRA database (accession BioProject: PRJNA928793).

## Abbreviations

*T. fortunei*: *Trachycarpus fortunei*
ABA: Abscisic acid
CCS: Circular consensus sequencing
CK: Cytokinin
DEG: Differentially expressed gene
FLNC: Full-length Non-concatemer
GH3: Gretchen Hagen 3
IAA: Indole-3-acetic acid
PCA: Principal component analysis
PacBio: Pacific BioSciences
PIF: PHYTOCHROME-INTERACTING FACTOR
PP2C: Protein phosphatase 2C
RIN: RNA integrity number
SAUR: Small auxin-up RNA
SMRT: Single-molecule real-time
SNP: Single nucleotide polymorphism
STEM: Short Time-series Expression Miner
STPK: Serine/threonine-protein kinase
TF: Transcription factor
TO-GCN: Time-ordered gene co-expression network
ZR: Trans-Zeatin-riboside
