# Supplementary material for: Comparative transcriptome analysis of dioecious floral development in Trachycarpus fortunei using Illumina and PacBio SMRT sequencing
Source: BMC Plant Biol. 2023 Nov 3;23:536. doi: 10.1186/s12870-023-04551-x (PMC10623883; doi:10.1186/s12870-023-04551-x)
Supplement: Supplementary file 2 — Additional file 2: Fig. S1. GO enrichment between different groups. A: Comparison between male and female plant combinations at the same time and position; B: Comparison between different part combinations of female plants; C:Comparison between different part combinations of male plants. Note: the size of the circle represented the gene count, the different colors represented the different groups; the fold enrichment equaled GeneRatio/BgRatio. Fig. S2. Sequence diagram of some hypothetical sex-related genes. Fig. S3. The systematic phylogenetic tree of all identified MADS-box [file 12870_2023_4551_MOESM2_ESM.docx]

**Supplementary Information:**


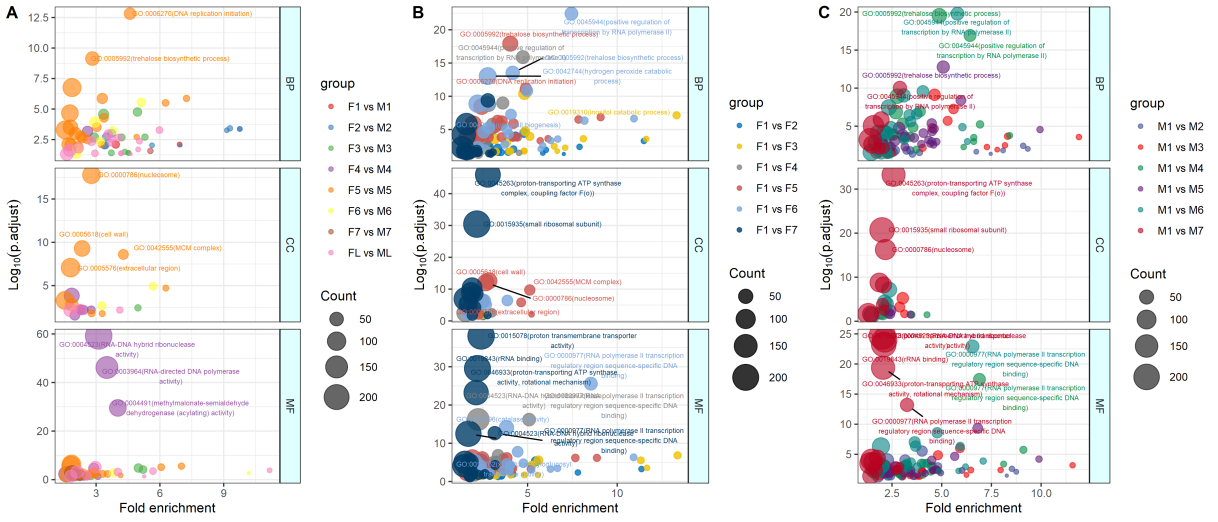


**Fig. S1.** GO enrichment between different groups. A: Comparison between male and female plant combinations at the same time and position; B: Comparison between different part combinations of female plants; C:Comparison between different part combinations of male plants. Note: the size of the circle represented the gene count, the different colors represented the different groups; the fold enrichment equaled GeneRatio/BgRatio.


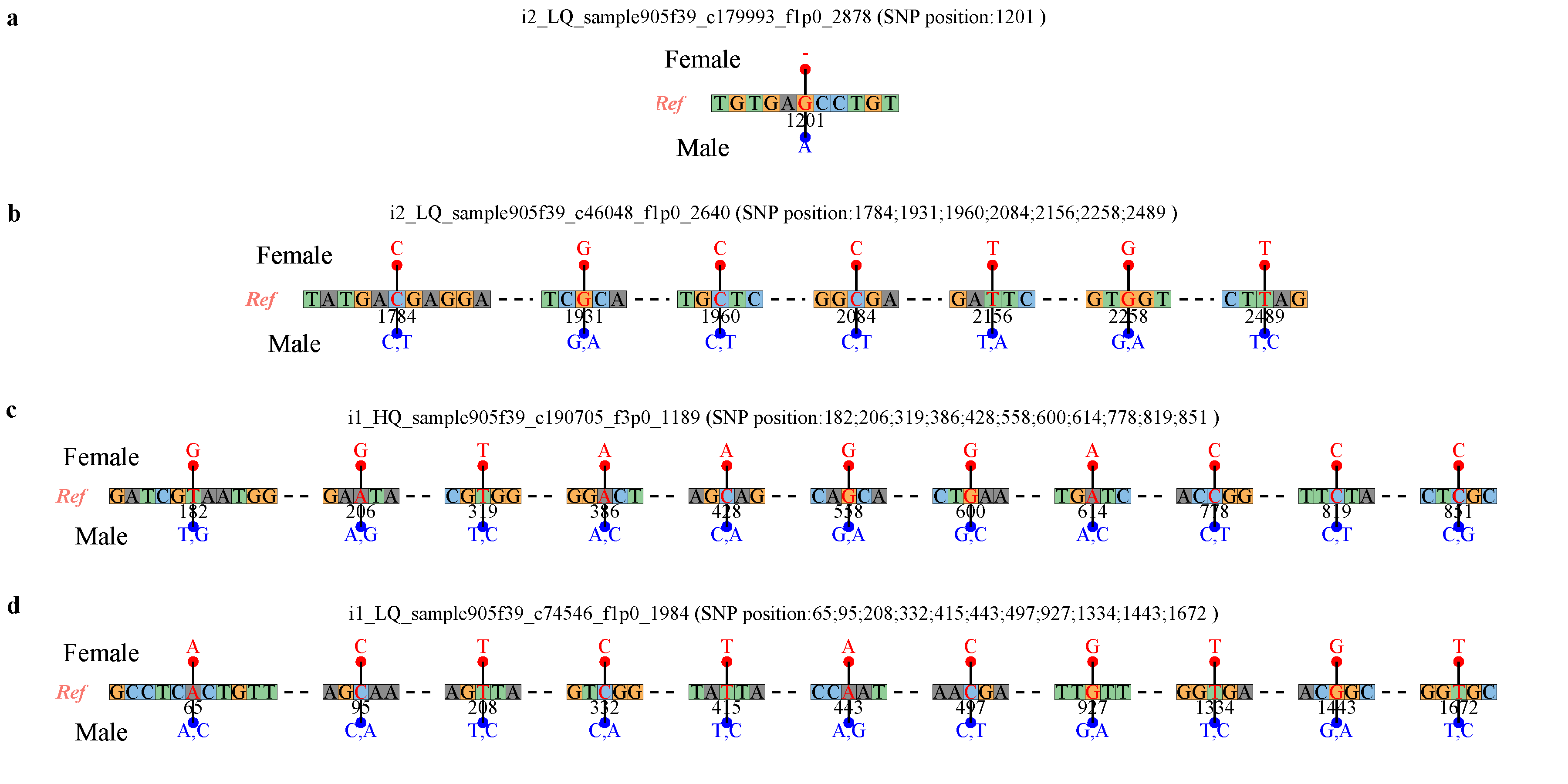


**Fig. S2.** Sequence diagram of some hypothetical sex-related genes.


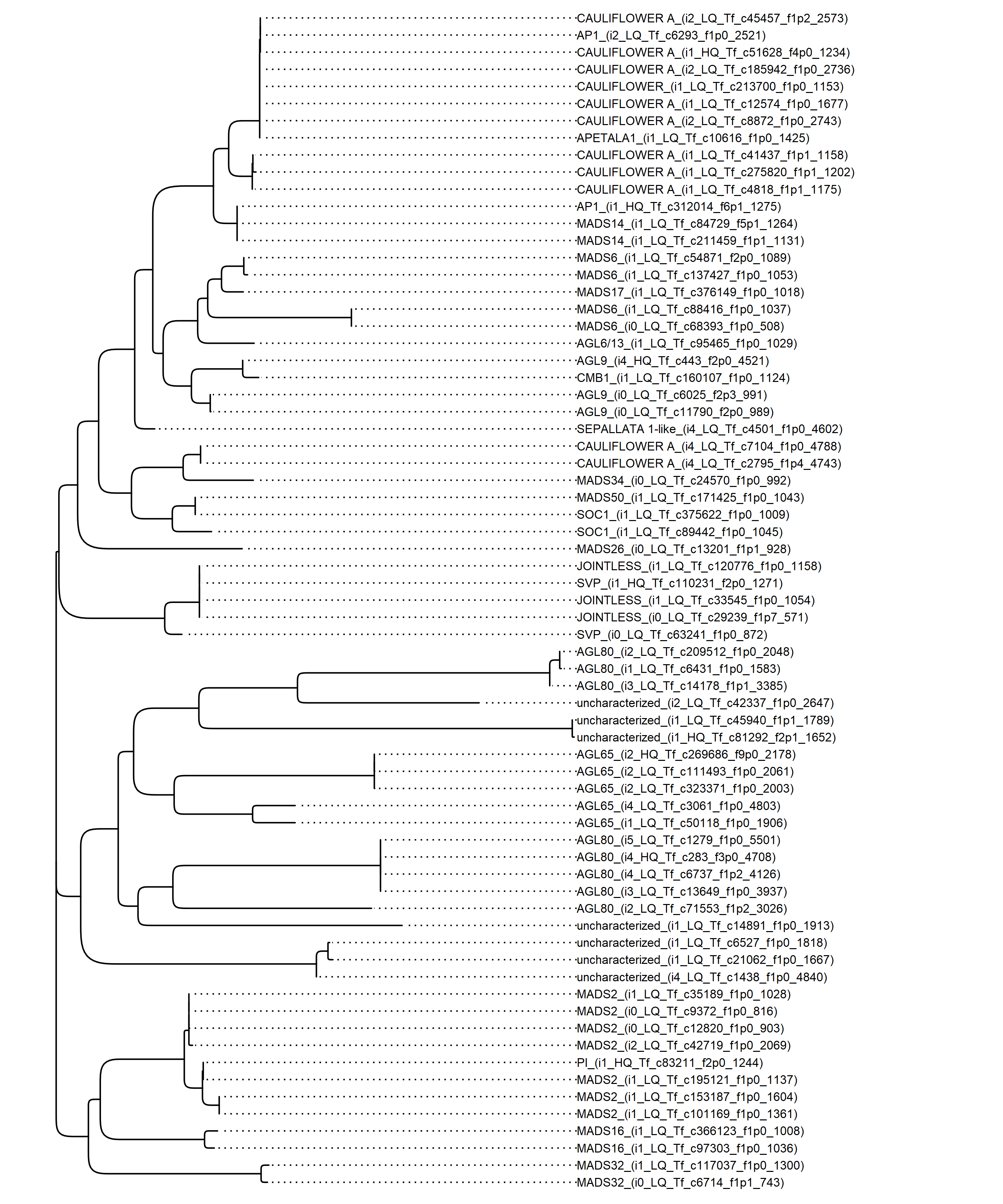


**Fig. S3.**The systematic phylogenetic tree of all identified MADS-box
